# Impaired Hepatitis C Virus-Specific T Cell Responses and Recurrent Hepatitis C Virus in HIV Coinfection

**DOI:** 10.1371/journal.pmed.0030492

**Published:** 2006-12-12

**Authors:** Arthur Y Kim, Julian Schulze zur Wiesch, Thomas Kuntzen, Joerg Timm, Daniel E Kaufmann, Jared E Duncan, Andrea M Jones, Alysse G Wurcel, Benjamin T Davis, Rajesh T Gandhi, Gregory K Robbins, Todd M Allen, Raymond T Chung, Georg M Lauer, Bruce D Walker

**Affiliations:** 1 Partners AIDS Research Center and Infectious Disease Division, Massachusetts General Hospital, Harvard Medical School, Boston, Massachusetts, United States of America; 2 Howard Hughes Medical Institute, Chevy Chase, Maryland, United States of America; 3 Lemuel Shattuck Hospital, Jamaica Plain, Massachusetts, United States of America; 4 Gastrointestinal Unit, Massachusetts General Hospital, Harvard Medical School, Boston, Massachusetts, United States of America; National Institutes of Health, United States of America

## Abstract

**Background:**

Hepatitis C virus (HCV)-specific T cell responses are critical for spontaneous resolution of HCV viremia. Here we examined the effect of a lymphotropic virus, HIV-1, on the ability of coinfected patients to maintain spontaneous control of HCV infection.

**Methods and Findings:**

We measured T cell responsiveness by lymphoproliferation and interferon-γ ELISPOT in a large cohort of HCV-infected individuals with and without HIV infection. Among 47 HCV/HIV-1-coinfected individuals, spontaneous control of HCV was associated with more frequent HCV-specific lymphoproliferative (LP) responses (35%) compared to coinfected persons who exhibited chronic HCV viremia (7%, *p* = 0.016), but less frequent compared to HCV controllers who were not HIV infected (86%, *p* = 0.003). Preservation of HCV-specific LP responses in coinfected individuals was associated with a higher nadir CD4 count (*r* ^2^ = 0.45, *p* < 0.001) and the presence and magnitude of the HCV-specific CD8^+^ T cell interferon-γ response (*p* = 0.0014). During long-term follow-up, recurrence of HCV viremia occurred in six of 25 coinfected individuals with prior control of HCV, but in 0 of 16 HIV-1-negative HCV controllers (*p* = 0.03, log rank test). In these six individuals with recurrent HCV viremia, the magnitude of HCV viremia following recurrence inversely correlated with the CD4 count at time of breakthrough (*r* = −0.94, *p* = 0.017).

**Conclusions:**

These results indicate that HIV infection impairs the immune response to HCV—including in persons who have cleared HCV infection—and that HIV-1-infected individuals with spontaneous control of HCV remain at significant risk for a second episode of HCV viremia. These findings highlight the need for repeat viral RNA testing of apparent controllers of HCV infection in the setting of HIV-1 coinfection and provide a possible explanation for the higher rate of HCV persistence observed in this population.

## Abstract 2

HIV infection impairs the immune response to HCV. Even individuals who have cleared HCV infection remain at significant risk for a second episode of HCV viremia.

## Editors' Summary

**Background.:**

Because of shared transmission routes (contaminated needles, contaminated blood products, and, to a lesser extent, unprotected sex), a large proportion of HIV-infected individuals (estimates range between 25% and 33%) are also infected with the hepatitis C virus (HCV). In most but not all individuals infected with HCV, the virus infection is chronic and causes liver disease that can eventually lead to liver failure. Disease progress is slow; it often takes decades until infected individuals develop serious liver disease. In people infected with both HCV and HIV, however, liver disease caused by HCV often appears sooner and progresses faster. As highly active antiretroviral therapy (HAART) and prophylaxis of opportunistic infections increase the life span of persons living with HIV, HCV-related liver disease has become a major cause of hospital admissions and deaths among HIV-infected persons.

**Why Was This Study Done?:**

A sizable minority of people who are infected with HCV manage to control the virus and never get liver disease, and scientists have found that these people somehow mounted a strong immune response against the hepatitis C virus. CD4^+^ T cells, the very immune cells that are infected and destroyed by HIV, play an important role in this immune response. The goal of the present study was to better understand how infection with HIV compromises the specific immune response to HCV and thereby the control of HCV disease progression.

**What Did the Researchers Do and Find?:**

The researchers recruited four groups of patients, 94 in total, all of whom were infected with HCV. Two groups comprised patients who were infected with HIV as well as HCV, with either high or undetectable levels of HCV (30 patients in each group). The two other groups included patients not infected with HIV, either with high or undetectable levels of HCV (17 patients in each group). The researchers focused on the individuals who, despite coinfection with HIV, were able to control their HCV infection. They found that those individuals managed to maintain relatively high levels of CD4^+^ T cells that specifically recognize HCV. However, a quarter of these patients (six out of 25) failed to keep HCV levels down for the entire observation period of up to 2.5 years; their blood levels of HCV rose substantially, most likely due to recurrence of the previously suppressed virus (the researchers could not be certain that none of the patients had become infected again after a new exposure to HCV-contaminated blood, but there was no evidence that they had engaged in risky behavior). The rise of HCV levels in the blood of the relapsed patients coincided with a drop in overall CD4^+^ T cell numbers. Following relapse in these individuals, HCV did not return to undetectable levels during the study. During the same period none of the 16 HIV-uninfected people with controlled HCV infection experienced a recurrence of detectable HCV.

**What Do These Findings Mean?:**

Despite the relatively small numbers of patients, these results suggest that recurrence of HCV after initial control of the virus is more likely in people who are coinfected with HIV, and that HCV control is lost when CD4^+^ T cell counts fall. This is one more reason to test all HIV-positive patients for HCV coinfection. Coinfected patients, even those who seem to be controlling HCV and would not automatically receive HCV treatment, should be regularly tested for a rise of HCV levels. In addition, maintaining CD4^+^ T cells at a high level might be particularly important for those patients, which means that doctors might consider starting HAART therapy earlier than is generally recommended for HIV-infected individuals. Additional studies are needed to support these recommendations, however, especially as this study did not follow the patients long enough to determine the consequences of the observed loss of control of HCV.

**Additional Information.:**

Please access these Web sites via the online version of this summary at http://dx.doi.org/10.1371/journal.pmed.0030492.

- AIDS Treatment Data Network factsheet on HIV/HCV coinfection
- US CDC factsheet on HIV/HCV coinfection
- American Liver Foundation, information on HIV and HCV
- MedlinePlus pages on HCV

## Introduction

Hepatitis C virus (HCV) infection is associated with persistence of viremia in the majority of infected persons, characterized by high plasma viral RNA titers with or without progressive liver disease. A minority of individuals establish long-term viral control and thereby do not experience disease progression, and spontaneous viral control is associated with broad and vigorous CD4^+^ T cell responses [1–3], which endure in the majority of persons [4]. In the United States and other countries, there is an increasing recognition of coinfection with HCV as a major problem in persons infected with HIV-1 [5]. Since the principal targets of HIV-1 infection are CD4^+^ T lymphocytes, two crucial questions are how infection with HIV impacts CD4^+^ T cell responses to pathogens such as HCV, and how this affects immune control of HCV infection.

For many acute viral infections, successful control is predicated on the generation and maintenance of long-lasting specific memory CD4^+^ T cells, which support the production of antibodies and the function of specific CD8^+^ T cells [6–9]. A rapidly growing body of evidence indicates that CD4^+^ T cell responses represent a critical component of a successful immune response against HCV [10]. During the acute phase of HCV infection, the breadth of this response correlates with early control [3,11]. After successful clearance, CD4^+^ T lymphocytes that proliferate in response to recognition of viral antigen are found in 64%–79% of spontaneous controllers [4,12], target multiple epitopes [13,14], and can outlast measurable humoral responses [4]. Recent CD4^+^ T cell depletion experiments in HCV-infected chimpanzees revealed the importance of these lymphocytes for the support of functional CD8^+^ T cell responses and the control of viremia [7]. In humans, CD4^+^ T cell depletion due to HIV is associated with declining levels of functional CD8^+^ T cells specific for HCV [9]. The above evidence suggests a critical role of virus-specific CD4^+^ T cells in HCV control.

Thus far, studies of the impact of HIV infection on HCV-specific CD4^+^ T cells have focused on persons with chronic HCV viremia [15–17]. The livers of HCV/HIV-coinfected individuals are depleted of total intrahepatic CD4^+^ T cells [18], correlating with the low levels of antigen-specific CD4^+^ T cell responsiveness within intrahepatic lymphocytes, even after long-term culture with interleukin-2 (IL-2) [17]. Analyses of peripheral blood in coinfected persons have similarly revealed a paucity of CD4^+^ T cell lymphocyte responses against HCV, whether measuring interferon-γ (IFN-γ) secretion [19,20] or proliferative capacity [15].

In this study, we focused on persons who are spontaneous controllers of HCV infection but are coinfected with HIV-1. We recruited a large cohort of HIV+ and HIV− participants, including 47 participants exhibiting evidence of spontaneous control of HCV, determined the effects of HIV-induced T cell depletion on CD4^+^ and CD8^+^ T cell responsiveness to both HIV-1 and HCV, and examined the durability of spontaneous control of HCV in the setting of HIV-1 coinfection.

## Methods

### Study Population

Participants were recruited from clinics of the Infectious Disease Division and Gastrointestinal Unit at Massachusetts General Hospital and Lemuel Shattuck Hospital, both in Boston, Massachusetts, United States. Participants gave written informed consent under protocols approved by the respective Institutional Review Boards and compliant with guidelines of the Declaration of Helsinki regarding protection of human subjects. Participants who had received prior interferon-based treatment for chronic HCV were excluded; therefore, HCV viral RNA levels represent the natural outcome of infection. In total, 94 participants were recruited who demonstrated HCV antibody by enzyme immunoassay, including 30 consecutively enrolled HIV+/HCV+ persons with HCV control, defined below. CD8^+^ T cell responses were reported in a separate study for the first 19 participants [9]. Nadir CD4^+^ T cell counts, defined as the historically lowest measured CD4^+^ T cell count, were determined via chart review; this information was unavailable for four participants. As one control group, 30 HIV+ participants with detectable HCV RNA were analyzed. In addition, 34 HCV-monoinfected participants, including 17 viremic participants and 17 controllers, were studied. All participants were analyzed after the early/acute phase of either HCV or HIV-1 infection (defined as >1 y from date of infection).

### Viral RNA Testing

Plasma HCV and HIV viral RNA titers were measured using the Cobas Amplicor Monitor assay 2.0 with a detection limit of 400 HIV-1 RNA copies/ml and 600 (2.8 log) HCV IU/ml (Roche Molecular Systems [http://www.roche-diagnostics.us]). Qualitative HCV viral RNA assays with a detection limit of 60 (1.8 log) IU/ml (Roche Molecular Systems) were utilized for confirmation of lack of viremia.

Spontaneous control of HCV was defined as less than 600 IU/ml on two consecutive plasma specimens in the absence of anti-HCV treatment and confirmed as less than 60 IU/ml by qualitative assay in 43 of 47 controllers of HCV in whom sufficient sample was available for retesting. Longitudinal viral RNA titers were obtained at 60–180 d intervals after first documentation of control of HCV; sustained control of HCV was defined as negative for all subsequent time points, including at least one confirmation by qualitative assay, whereas HCV recurrence was defined as previous spontaneous HCV control with subsequent viremia on consecutive time points, including both possibilities of recrudescent infection (return of original infecting virus) or reinfection (new infection). The timing of recurrence was calculated as the point halfway between the last negative measurement and the first positive measurement.

### Peptides and Recombinant Antigens

Recombinant protein HIV p24 (Protein Sciences [http://www.proteinsciences.com]) and CMV viral lysate (BioWhittaker [http://www.cambrex.com]) were obtained commercially with appropriate controls. HCV proteins were expressed as carboxy-terminal fusion proteins with human superoxide dismutase in Saccharomyces cerevisiae or Escherichia coli (Chiron [http://www.chiron.com]). Individual proteins were derived from the HCV-1 sequence and encoded core (c22–3 aa 2–120), NS3 (c33c aa 1192–1457), NS4 (C100.3 aa 1569–1931), and NS5 (aa 2054–2995). Two overlapping peptide sets corresponding to the HCV-1 and HCV-H77 1a full HCV genomes were also utilized as described previously [9,13].

### Lymphoproliferative Assay

Lymphoproliferative (LP) assays were performed as previously described using designated viral antigens and corresponding controls (superoxide dismutase, *E. coli,* or the baculovirus-derived control mgs) at concentrations of 10 μg/ml [13,15,21]. Fresh peripheral blood mononuclear cells (PBMCs) were isolated from blood obtained in heparin, trisodium citrate, citric acid, or dextrose tubes by Ficoll-Hypaque density gradient centrifugation and plated at 100,000 cells/well in 96-well U-bottom plates (Costar [http://www.costar.com]) in 200 μl of R10/Hab medium (i.e., RPMI 1640, 10% human antibody serum; both Sigma-Aldrich [http://www.sigmaaldrich.com]) and 10 mM HEPES buffer (Sigma-Aldrich) with 2 mM glutamine and antibiotics (penicillin and streptomycin, 50 U/ml). Each protein and each corresponding control was placed in quadruplicate wells for each assay. After 6 d at 37 °C and 5% CO_2_, wells were pulsed for 6 h with 1.0 μCi of ^3^H thymidine (NEN [http://las.perkinelmer.com]). Cells were collected on filters, and incorporated radiolabel was measured with a beta counter (Packard Topcount, Packard Instruments [http://las.perkinelmer.com]).

The stimulation index (SI) was defined as the ratio of the mean counts per min of the antigen wells to the mean counts per minute of the control protein wells. For the purposes of data interpretation, SI of five or more was considered significant [13,15,21]. For cross-sectional analysis, the first time point tested was utilized. Only assays with a positive response to the phytohemagglutinin control were included.

### Antigen-Stimulated Cell Lines

Virus-specific CD4^+^ T cell lines were generated via stimulation of fresh PBMCs with R10 medium supplemented with recombinant IL-2 (50 U/ml) and the respective recombinant HCV antigen (1 μg/ml) [13,14] or by the appropriate peptide as previously described [9,22]. These cell lines were tested two to four weeks after stimulation.

### Enzyme-Linked Immunospot for Single-Cell Quantification of IFN-γ Secretion

Enzyme-linked immunospot (ELISPOT) assays were performed exactly as previously described [9]. Whether a specific response was CD8^+^ or CD4^+^ was determined by depletion using magnetic beads and columns according to the manufacturer's instructions (Miltenyi Biotec [http://www.miltenyibiotec.com]) and/or testing by ELISPOT and intracellular cytokine staining on short-term cell lines in the presence of IL-2.

### Statistical Analysis

Fisher's exact test was used for comparison of proportions. For cross-sectional analyses, nonparametric comparisons were used for intergroup comparisons, and Spearman correlations or linear regressions performed for determining the relationship between continuous variables and immunologic parameters. Kaplan-Meier analysis was used to describe times to viral recurrence, difference between groups compared by the log rank test; data were censored at the last available time point. The degree of control of viremia was calculated as area under the curve during the year following recurrence. Graphical representations and statistical analyses were performed using Microsoft Excel and GraphPad Prism 3.0 (GraphPad Software [http://www.graphpad.com]).

## Results

### Clinical Characteristics of Participants Exhibiting Spontaneous Control of HCV

To define the impact of superimposed HIV infection on the ability to control HCV viremia, we established four groups of participants, all HCV antibody positive. Those with HCV monoinfection were divided into those who have persistent viremia and those who spontaneously control viremia without anti-HCV therapy. Similarly, those coinfected with HIV were divided between one group consisting of individuals with persistent HCV viremia and another group exhibiting spontaneous control of HCV. Summary clinical data regarding all patient groups are presented in Table 1.

**Table 1:**
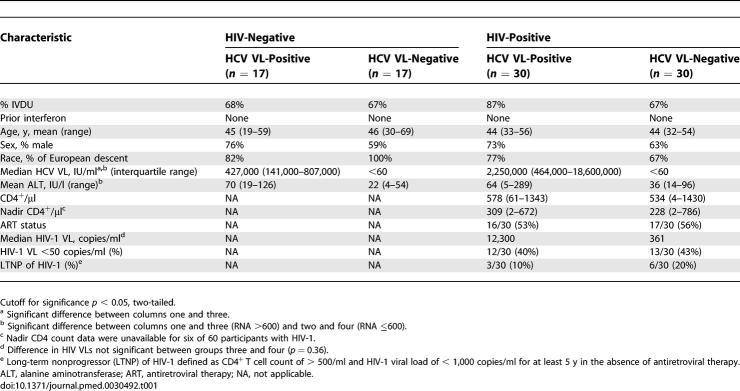
Characteristics of 94 HCV-Infected Patients

When comparing participants who spontaneously control HCV with those who do not, there were no significant differences in demographic parameters such as age, race, or sex. The only clinical, demographic, or laboratory parameter associated with HCV spontaneous control was normal alanine aminotransferase levels (Table 1, *p* = 0.001). In dually infected persons with and without HCV viremia, there were no differences in CD4^+^ count or antiretroviral use. A decrease in HIV viral loads (VLs) was detected in HIV+ persons with spontaneous HCV control, although it did not reach statistical significance (median HIV VL, HCV chronic, 12,300 copies/ml; HCV controller 361 copies/ml; *p* = 0.36, Fisher's exact test). We found a similar proportion of persons who control HIV-1 (defined as < 1,000 copies/ml in the absence of antiretroviral treatment) in the HCV viremic and HCV aviremic groups, indicating that spontaneous control of HCV in this cohort did not correlate with control of HIV-1 within the same individual, although larger studies with greater power are needed to address this relationship.

### Multispecific CD4^+^ LP Responses are Linked to Spontaneous Control of HCV in HIV+ Persons

**Figure 1:**
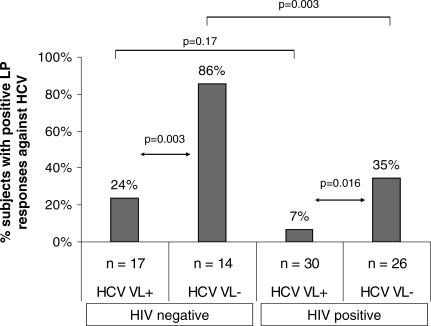
Rate of Detection of LP Responses against HCV Antigens by Subgroup, Stratified by HIV Status and HCV Viremia *p*-Values represent comparison of proportions per Fisher's exact test. HIV− persons with spontaneous control of viremia had the highest rate of detection (86%). By comparison, HIV+ persons with spontaneous control of viremia exhibited a 35% rate of LP responses. Both groups of controllers demonstrated significantly higher rates of detection than did their viremic counterparts. Fresh cells were not available for analysis of four of 30 HIV-1/HCV controllers.

**Figure 2:**
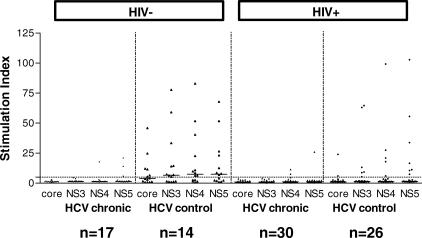
LP Responses to HCV Antigens Are Associated with Spontaneous Control of Viremia Regardless of HIV Status Stimulation indices to HCV proteins are plotted per subgroup. The two plots on the left represent responses in HIV− persons; the two on the right in HIV+. Cutoff of significance for lymphoproliferation was an SI of 5, shown by horizontal dotted line. Median values are also shown.

CD4^+^ T cell responsiveness in these groups, subdivided by HIV-1 status and HCV VL, was compared using a 2 × 2 factorial design. We compared HCV-specific CD4^+^ LP responses between monoinfected and coinfected participants, since these responses correlate with successful immunity against HCV [1,4,12]. In monoinfected participants, spontaneous control of HCV was strongly associated with proliferative responses (HCV controller, 12 of 14 [86%], versus HCV viremic, 4 of 17 [24%]; *p* = 0.003, Fisher's exact test) (Figure 1), a finding consistent with prior studies [4,12,13]. In the presence of dual infection with HIV, HCV-specific responses were enriched in HCV spontaneous controllers, but HIV had a clear deleterious effect in that a significantly smaller percentage of persons had these responses when compared to those with monoinfection (HIV-1/HCV controllers, 9 of 26 [35%], versus HIV+/HCV+ chronics, 2 of 30 [7%]; *p* = 0.016, Fisher's exact test) (Figures 1 and 2). In both HIV+ and HIV− controllers of HCV, responses against HCV were often multispecific within single individuals (16 of 21 positive assays among HCV controllers, unpublished data).

**Figure 3:**
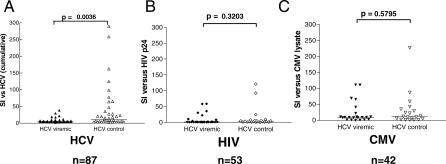
HCV Viremia Is Associated with Decreased Lymphoproliferation against HCV Antigens But Not against HIV or CMV (A) The cumulative SI against HCV antigens core, NS3, NS4, and NS5. Four of 60 coinfected participants and three of 34 monoinfected participants could not be tested due to lack of fresh PBMCs, thus 87 of 94 of the total cohort were tested. (B) Responses against HIV-1 p24 antigen. Of 60 coinfected participants, 53 were tested. (C) Responses against CMV in coinfected participants, including the 42 participants confirmed as seropositive for CMV.

We next examined whether the lack of responsiveness to HCV antigens in the viremic group was due to a specific effect of viremia on HCV-specific responses or due to a general inhibition of CD4^+^ lymphoproliferation. The cumulative SI to HCV antigens was significantly higher in those with spontaneous control of HCV (*p* = 0.004; Mann-Whitney) (Figure 3A); noting that fresh cells were not available to test seven participants in the overall cohort. In coinfected participants, we examined LP responses to HIV p24 and CMV, limiting our analysis to participants seropositive for each respective infection. We detected no abrogation of responses to either p24 or CMV in the HCV viremic group compared to those with HCV control (HIV, *p* = 0.32; CMV, *p* = 0.58; Mann-Whitney) (Figure 3B and 3C). There was also no significant difference between responses to phytohemagglutinin between the two groups (unpublished data). In participants not on antiretroviral therapy, LP responses to p24 antigen inversely correlated with HIV VLs (*n* = 18, *r* = −0.72, *p* ≤ 0.001, Spearman) consistent with prior studies [21,23,24]. These data suggest that HCV viremia is associated with a selective loss or impairment of HCV-specific CD4^+^ T cells rather than a global impairment of T cell proliferation to other stimuli.

### A Higher Nadir CD4^+^ T Cell Count Correlates with Preserved HCV-Specific CD4^+^ T Cell Responses against HCV in Dually Infected Participants

**Figure 4:**
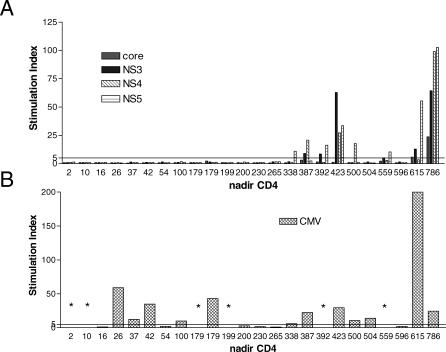
LP Responses in HIV+ Participants Who Spontaneously Control HCV No participant with a history of CD4^+^ T cell depletion (nadir CD4 < 300 cell/μl) displayed LP response to HCV antigens (A), contrasting with responses to CMV in which responses were detected in individuals with a history of significant CD4^+^ T cell depletion (B). Those marked with * are CMV−, and values are therefore not shown

Since the above data indicate that HCV-specific LP responses are less frequently detected in persons dually infected with HIV-1, we investigated whether contemporaneous or historical nadir CD4^+^ T cell counts were predictive of LP responses against HCV in HIV-1-coinfected persons with control of HCV. Since the primary pathogenic outcome of HIV-1 infection is progressive loss of CD4^+^ T cells, both contemporaneous and historical nadir T cell counts are potential predictors of LP responses against HCV in this subgroup. HCV-specific CD4^+^ LP responses were strongly correlated with nadir CD4^+^ counts but not with contemporaneous counts for each HCV antigen (Table 2). Responses against HIV p24 antigen followed a similar pattern as HCV, in contrast to responses against CMV and responses following stimulation with phytohemagglutinin, which showed no significant correlation (Table 2). When participants with a history of either earlier treatment (nadir CD4^+^ T cell count >300) or long-term spontaneous control of HIV were combined (Figure 4), HCV-specific responses resembled the HCV monoinfected controllers in terms of breadth and magnitude.

**Table 2:**
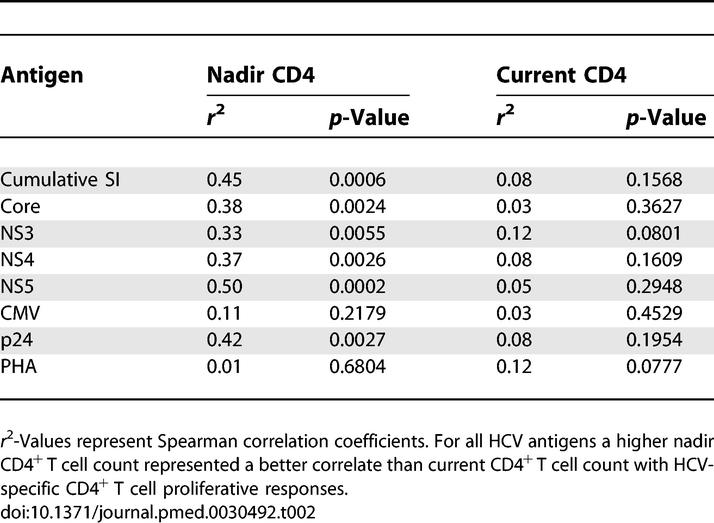
LP Responses to HCV Antigens in HIV+ HCV Controllers Correlate More Strongly with Historical Nadir CD4^+^ T Cell Counts Than with Contemporaneous CD4^+^ T Cell Counts

We also performed parallel assays in the presence of the antiretroviral drug zidovudine in vitro (0.5 μM) and after CD8^+^ T cell depletion with magnetic beads and were unable to enhance our detection of LP responses against HCV (unpublished data). These results suggest that the relative paucity of proliferating HCV-specific CD4^+^ T cells in HIV+ persons who maintain HCV control is not due to HIV-1 replication in the culture or to CD8-mediated suppressive effects.

### Relationship of HCV-Specific CD4^+^ LP Responses to IFN-γ T Cell Responses

**Figure 5:**
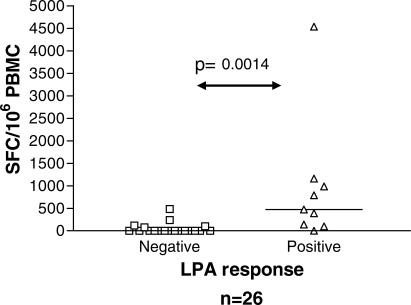
Relationship of CD4^+^ T cell Proliferation against HCV Antigens to CD8^+^ IFN-γ Responses Total CD8^+^ IFN-γ response was determined via a comprehensive ELISpot assay against the entire HCV-expressed polyprotein. In HIV+ participants who control HCV spontaneously, the magnitude of IFN-γ responses mediated by CD8^+^ T cells is significantly higher in participants with LP responses against HCV (*p* = 0.0014, Wilcoxon rank-sum test).

Having demonstrated that dual infection with HIV is associated with lower frequencies of HCV-specific CD4^+^ T cell responses, we determined whether this effect has an impact on the magnitude of HCV-specific CD8^+^ T cell responses. The ex vivo CD8^+^ IFN-γ response to a comprehensive panel of antigens at the same time point was determined by overnight ELISpot, using methods as previously described [9]. IFN-γ-producing CD8^+^ T cells were found to be present at higher magnitudes in those with CD4^+^ LP responses (*p* = 0.0014, Mann-Whitney) (Figure 5).

### Recurrence of HCV Viremia in HIV+ Participants with Prior Control of HCV

**Figure 6:**
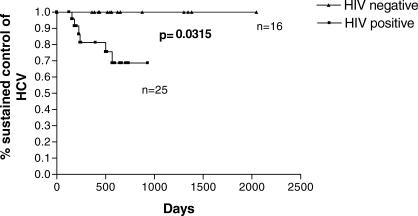
Recurrent Viremia in Persons with Evidence of Spontaneous Control of HCV Serial HCV VL determinations were performed in persons demonstrating spontaneous control of HCV. Data were censored at the last available time point. Six of 25 participants experienced recurrent HCV viremia in the HIV+ group, while 0 of 16 HIV− participants experienced recurrence. The median time of follow-up did not differ between groups.

Recurrence of viremia following clearance of HCV infection has been reported in monoinfected persons who previously cleared viremia spontaneously [25,26]. We examined the durability of viral control in participants with spontaneous HCV control with and without HIV-1. We obtained repeat HCV viral RNA testing at 60–180 d intervals in 25 individuals (median length of follow-up was 438 d, range 121–932). A total of 19 coinfected participants maintained undetectable HCV RNA, but six experienced recurrent viremia (Table 3). Repeat viral RNA testing of 16 HIV-1-negative HCV controllers revealed no instances of recurrent viremia despite a similar number of measurements and lengths of follow-up (median follow-up 518 d, range 361–2,047 d). By Kaplan-Meier analysis, recurrent viremia was more likely to occur in the HIV-1-positive group, suggesting that those with HIV coinfection are at risk for these events (*p* = 0.03, log rank test) (Figure 6). Comparing responses, we detected a trend towards lower magnitude (cumulative SI to all HCV antigens) of LP responses in those who experienced recurrence versus all controllers who did not (*p* = 0.08, Mann-Whitney).

**Table 3:**
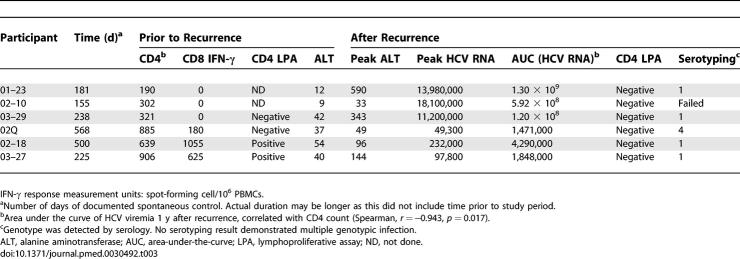
Clinical Data and Outcomes of Six HCV Recurrences in HIV+ Persons with Prior Control of HCV

Lack of samples from the acute phase of infection and inability to sequence virus from periods of viral control prevented determination of whether recurrent viremia represented loss of intrinsic control of virus, which might have been facilitated by the diminished HCV-specific CD4^+^ proliferative responses associated with HIV-1 or reinfection with a novel strain [25]. Results of serotyping experiments on plasma from these episodes of recurrence did not reveal evidence of infection with more than one genotype that would implicate reinfection as the primary mechanism (Table 3). None of the six individuals reported ongoing high-risk behavior, especially active intravenous drug use, the most efficient method of transmission to support the latter hypothesis. Two of the six participants were never intravenous drug users (IDUs) (routes of transmission were sexual and related to blood products); the other four were former IDUs.

### Relevance of Preexisting Immune Status and Responses to Viral Recurrence

We next examined the clinical outcomes of these six recurrences of HCV viremia in HIV-1-infected individuals with prior control of HCV. None of six participants who experienced recurrence regained sustained control of HCV; instead, persistent viremia was established in each case. Furthermore, none of the six participants had symptoms consistent with acute illness at the time of viral recurrence, and only three of the six participants experienced significant (>3 × upper limit of normal range) elevations in alanine aminotransferase (Table 3). Wide ranges of HCV viral RNA titers were observed following recurrence (HCV RNA range, 141–1.8 × 10^7^ IU/ml) (Table 3).

We also examined the relationship between immune status and magnitude of HCV viremia following recurrence. Although the number of study participants is small, we observed that the CD4^+^ T cell count at time of breakthrough negatively correlated with the calculated area-under-the-curve of viremia (*r* = −0.943, *p* = 0.017, Spearman). These data suggest that general immune status and/or HCV-specific immunity have an impact on subsequent viral control after recurrence.

**Figure 7:**
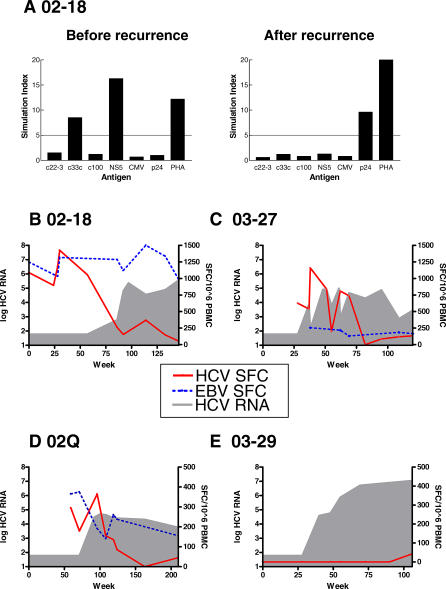
HCV-Specific T Cell Responses before and after HCV Recurrence in HIV+ Participants with Prior Spontaneous Control (A) LP responses before and after HCV recurrence. Responses to c33c (NS3) and NS5 declined after the establishment of viremia. By contrast, stimulation indices to HIV-1 p24 antigen and phytohemagglutinin (positive control) increased. (B–E) The degree of viremia is shown in shaded gray for four participants. IFN-γ measurements are in spot-forming cell (SFC)/10^6^ PBMCs. The cumulative HCV CD8^+^ T cell response is represented by the solid red lines. In three participants who demonstrated decline in HCV-specific CD8^+^ T cell responses, Epstein–Barr virus (EBV) response to the lytic A2 epitope (GLCTLVAML) is shown by the blue dashed lines.

For five participants (02–18, 03–27, 02Q, 03–29, and 01–23), we quantified T cell responses before and after recurrence. In participants 02–18 and 03–27, CD4^+^ LP responses against HCV overall declined following recurrence (data for 02–18 shown in Figure 7A) (Table 3). In participants 02–18, 03–27, and 02Q, who demonstrated significant preexisting CD8^+^ IFN-γ responses and lower degree of viremia following recurrence (Table 3), the magnitude of HCV-specific CD8+ T cell responses over time declined, in contrast to a response to an immunodominant Epstein–Barr virus epitope (Figure 7B–7D). By contrast, when we analyzed two participants with very high levels of viremia following recurrence (Table 3), participant 01–23 exhibited no detectable ex vivo ELISpot response prior to or following viral recurrence (unpublished data). Participant 03–29 had no detectable response by either LP or ELISpot measurement prior to viral recurrence, and we measured a single weak CD8^+^ T cell response (50 spot-forming cells/10^6^ PBMCs) at the cutoff for significance at only one of five time points following recurrence (Figure 7E). While HCV-specific CD4^+^ T cell lines were expanded easily from time points of viral control in two of these participants, following recurrent viremia we were unable to expand cells despite IL-2 supplementation, either by using individual peptides corresponding with previously known CD4 specificities or by whole antigen stimulation (unpublished data). In summary, individuals with higher CD4^+^ T cell counts, and therefore preserved immunity, exhibited lower levels of HCV viremia following recurrence, and the shift from controlled to chronic HCV viremia was associated with attenuation of circulating immune responses.

## Discussion

Although spontaneous control of HCV infection is associated with long-lasting cell-mediated immune responses [3,4,13,23] and with partial protection following rechallenge [25], the role of cell-mediated immune responses and their relation to durability of control in the context of HIV infection remain unclear. Here we analyzed the impact of HIV infection on this apparent immune control in persons who spontaneously control HCV infection with coexisting HIV. We show that CD4^+^ T cell depletion induced by HIV-1 is associated with loss of adaptive HCV-specific immune responses. In addition, our findings indicate that: (1) HIV-infected individuals with spontaneous control of HCV maintained vigorous CD4^+^ T cell responses against HCV, if they exhibited higher nadir CD4^+^ T cell counts; (2) additional episodes of HCV viremia occur frequently in HIV+ participants relative to their HIV− counterparts; (3) preservation of higher CD4^+^ T cell counts, and therefore HCV immunity, is potentially relevant to subsequent HCV viral RNA levels following recurrence.

In HCV monoinfection, the vast majority of participants who spontaneously control HCV generate [27] and maintain [1,4,12,13] vigorous peripheral LP responses to HCV antigens. Our previous study in 17 HIV-1/HCV-coinfected individuals found no LP responses against HCV [15], consistent with studies indicating that HCV-specific CD4^+^ T cell responses are absent or at strikingly low frequency in the setting of HIV-1 coinfection [17,20,28]. However, by focusing on HIV/HCV-coinfected individuals who demonstrated spontaneous control of HCV, we identified certain HIV-1-infected individuals with preserved circulating HCV-specific CD4^+^ T cells. In these individuals, CD4^+^ lymphocytes target multiple proteins and specificities as has been shown for the monoinfected population [13,14]. Maintenance of higher CD4^+^ T cell counts, either through long-term nonprogression of HIV or through antiretroviral treatment before CD4^+^ T cell counts fell below ∼300 cells per microliter, was the most important shared feature of these individuals. Conversely, we observed that participants with low nadir CD4^+^ T cell counts, but significant immune reconstitution on antiretroviral therapy, did not recover HCV-specific CD4^+^ LP responses, accounting for why nadir was a better predictor than contemporaneous CD4^+^ T cell count. Since we did not detect HCV responses in those with nadir CD4^+^ T cell counts below 300, in contrast to those against CMV, our data suggest that the threshold CD4^+^ T cell count that results in loss of adaptive responses may be higher for HCV-specific responses than those to CMV [29]. As a corollary, these data suggest that treatment of HIV infection before advanced immunosuppression may preserve memory T cell responses against HCV.

What, then, is the importance of maintaining these responses? HCV-specific CD4^+^ T cell responses may confer benefits, as they may or may not be associated with improved liver histology in chronically infected participants [20] and are a crucial component of protective immunity upon rechallenge in the chimpanzee model, by playing a key role in supporting the pool of circulating functional CD8+ T cells [7]. Our study was not designed to examine the former concept but provides evidence that supports the latter, as the viral RNA levels observed in our coinfected participants with recurrence were much higher than those from the earlier study of high-risk IDUs [25].

Our results also show a high incidence (24%) of recurrent HCV viremia in HIV-1-coinfected participants with initial control of HCV, even in some who had previously maintained HCV control for almost two years of observation. Whether the cases of recurrent HCV viremia described in this report are due to recrudescence of initially controlled virus [26,30] or reinfection with a newly acquired strain remains unanswered by the present study. Loss of control of HCV viremia has been described in both chimpanzee and human models within the year following acute infection [1,3], however, the six participants in the present study experienced recurrent viremia long after the acute phase. In addition, our interviews of participants and providers indicate a lack of active risk factors for new exposure to virus, although we cannot rule out this possibility definitively due to the potential of underreporting of these risk factors and/or susceptibility to other means of HCV transmission.

Regardless of whether our cases represent reinfection or recrudescence, loss of adaptive immunity and susceptibility to recurrent viremia provide potential explanations why HIV coinfection abrogates protective immunity to HCV and is associated with a higher rate of persistent HCV [31,32]. In most coinfected patients, HCV infection precedes HIV infection due to its greater transmissibility by blood, and the outcome of HCV should be determined prior to acquisition of HIV. Unlike the reinfections detected in those with history of prior viral control in the study by Mehta and colleagues [25], none of our participants with HCV recurrence were able to reestablish control, and those with the least immunity established extremely high levels of HCV viremia. Although limited by sample size, our data indicate that the level of preserved immunity prior to recurrence has an impact on the subsequent degree of HCV viremia following a second challenge to the immune system, consistent with findings from chimpanzee studies [7,33] and with the concept of secondary immunity against HCV [25].

This study highlights the need for repeat HCV RNA testing for persons with prior spontaneous control and suggests that HIV-1 is a cofactor for recurrent HCV viremia [34]. The clinical relevance of the observed viral recurrences remains unclear, but the shift from sustained control to chronicity likely confers risk of liver disease progression, which is accelerated in HIV-1-infected persons [5]. Moreover, our cross-sectional and longitudinal data extend other studies in monoinfection that show that HCV chronicity is associated with an attenuation of specific responses in the periphery [4,12,13]. This decline of responses may have preceded HCV viremia; alternatively, persistent HCV viremia may have altered HCV-specific responses through chronic antigenic stimulation.

In both our cross-sectional and longitudinal studies, the high circulating viral titers of HCV in persistently viremic individuals were not associated with a global change in responses against other herpes viruses, indicating that HCV viremia is associated with a lack of HCV-specific CD4^+^ T cells but does not significantly inhibit CD4^+^ T cell proliferation to other viral antigens. The decrement of functional responses in the presence of viremia may be due to a variety of mechanisms, including viral escape, T cell deletion or exhaustion, and compartmentalization. The hypothesis that loss of virus-specific CD4^+^ T cell responses associated with chronic HCV infection results in loss of CD8^+^ T cell responses and viral escape [7] deserves further study in coinfected humans. Also, while proliferative capacity is a strong correlate of viral control, it has been suggested that other functions of T cells may not correlate with proliferation [35,36]; therefore, further detailed characterization of these cells should elucidate further mechanisms of loss of viral control. Finally, we provide support of the concept that therapeutic vaccines or immunotherapies that successfully restore the frequencies and function of HCV-specific CD4^+^ T cell responses in chronically infected individuals may result in improved control. This may be particularly important in individuals dually infected with HIV-1, whose high HCV titers interfere with efficacy of interferon-based treatments [37].

In summary, we provide a detailed analysis of HCV-specific CD4^+^ T cells in the unique setting of HIV-1-infected participants with spontaneous control of HCV. T cell responses against HCV are preserved by avoiding CD4^+^ T cell depletion due to progressive HIV-1 infection, indicating a potential immunologic benefit of treatment of HIV in coinfected individuals before CD4^+^ T cell counts fall below 300 cells per microliter, a threshold at the higher end of the current range where initiation of treatment is typically recommended. The importance of preserving these adaptive immune responses is implied by: (1) the observed loss of control of HCV only among HIV-1-infected individuals with prior clearance of HCV and not in HIV− counterparts; and (2) their relevance to intrinsic control of HCV following rechallenge. Further understanding of the immune correlates of maintaining natural HCV control and subsequent loss of control will enhance our knowledge of the quality and quantity of CD4^+^ and CD8^+^ T cell responses necessary to elicit long-lasting and cross-protective immunity.

## Abbreviations

ELISPOT: enzyme-linked immunospot
HCV: hepatitis C virus
ICS: intracellular cytokine staining
IDU: intravenous drug user
IFN-γ: interferon-γ
IL-2: interleukin-2
LP: lymphoproliferative
PBMC: peripheral blood mononuclear cell
SI: stimulation index
